# Global Distribution of *Culex tritaeniorhynchus* and Impact Factors

**DOI:** 10.3390/ijerph20064701

**Published:** 2023-03-07

**Authors:** Yixin Tong, Honglin Jiang, Ning Xu, Zhengzhong Wang, Ying Xiong, Jiangfan Yin, Junhui Huang, Yue Chen, Qingwu Jiang, Yibiao Zhou

**Affiliations:** 1 School of Public Health, Fudan University, Building 8, 130 Dong’an Road, Shanghai 200032, China; 2 Key Laboratory of Public Health Safety, Fudan University, Ministry of Education, Building 8, 130 Dong’an Road, Shanghai 200032, China; 3 Center for Tropical Disease Research, Fudan University, Building 8, 130 Dong’an Road, Shanghai 200032, China; 4 School of Epidemiology and Public Health, Faculty of Medicine, University of Ottawa, 600 Peter Morand Crescent, Ottawa, ON K1G 5Z3, Canada

**Keywords:** *Culex tritaeniorhynchus*, global distribution, species distribution models, impact factors, climate change, habitat suitability, extension

## Abstract

Culex tritaeniorhynchus is the primary vector of Japanese encephalitis (JE) and has a wide global distribution. However, the current and future geographic distribution maps of *Cx. tritaeniorhynchus* in global are still incomplete. Our study aims to predict the potential distribution of *Cx. tritaeniorhynchus* in current and future conditions to provide a guideline for the formation and implementation of vector control strategies all over the world. We collected and screened the information on the occurrence of *Cx. tritaeniorhynchus* by searching the literature and online databases and used ten algorithms to investigate its global distribution and impact factors. *Cx. tritaeniorhynchus* had been detected in 41 countries from 5 continents. The final ensemble model (TSS = 0.864 and AUC = 0.982) indicated that human footprint was the most important factor for the occurrence of *Cx. tritaeniorhynchus*. The tropics and subtropics, including southeastern Asia, Central Africa, southeastern North America and eastern South America, showed high habitat suitability for *Cx. tritaeniorhynchus*. *Cx. tritaeniorhynchus* is predicted to have a wider distribution in all the continents, especially in Western Europe and South America in the future under two extreme emission scenarios (SSP5-8.5 and SSP1-2.6). Targeted strategies for the control and prevention of *Cx. tritaeniorhynchus* should be further strengthened.

## 1. Background

Japanese encephalitis (JE) is caused by infection with the Japanese encephalitis virus (JEV) with an overall incidence of 1.8 per 100,000 [1,2,3,4]. Among them, 20–30% are fatal, and 30–50% of the survivors have a poor prognosis [5]. *Cx. tritaeniorhynchus* is the primary vector of JEV [6] and is widespread in Southeast Asia, the Middle East, Africa and Europe [7]. There are some other human and animal viral diseases that are also transmitted by *Cx. tritaeniorhynchus*, including Dengue fever, Rift Valley fever and Tembusu virus infection fever [8,9,10]. *Cx. tritaeniorhynchus* breeds mainly in paddy fields [11] and shows a large expansion of geographic distribution and magnitude in many countries because of the development of rice agriculture [12,13,14]. Pigs and cattle show an intimate connection with the abundance of *Cx. tritaeniorhynchus* [9,15,16,17,18,19]. Climate and altitude also influence the distribution of *Cx. tritaeniorhynchus* [20,21]. Although predictive studies have paid attention to the various factors including climate and altitude [22], these results are not sufficient enough to distinguish between areas where the species can and cannot survive. Human footprint, as a composite indicator of human impact, including human population density, land transformation, human access and infrastructures, is often used in predicting species distribution [23]. In addition, Liu et al. reported that human impact is a strong predictor of *Cx. tritaeniorhynchus* [24]. Considering the importance of impact factors in prediction, it is still necessary for us to make integration and inclusion with more comprehensive and extensive factors.

Applying various methods to model the spatial distribution of vector species can assist in the assessment and management of associated health risks [25]. Different modeling techniques are successfully used to investigate potential geographical distribution in multifarious species, such as *Culicoides imicola*, *Aedes aegypti*, *Biomphalaria straminea* and *Oriental beech* [26,27,28,29]. Though previous modeling efforts obtained the current distribution maps of *Cx. tritaeniorhynchus* in China, Vietnam, Iran, Korea and even Asia [7,24,30,31,32], the current and future global geographic distribution maps are still missing. Especially in recent years, *Cx. tritaeniorhynchus* has also spread further worldwide with the clear trend of population expansion [33,34]. These signs call for more comprehensive and in-depth research to fill the gaps in the field, especially beyond Asia, to the global and future projections of *Cx. tritaeniorhynchus.*

Plentiful species distribution models (SDMs) are effectively used to predict species distributions using species occurrence and impact factors, especially for species expansion, by quantifying the mathematical and physical relationships between species and environmental variables, including a dozen of algorithms [28,35,36,37]. By identifying non-random relationships, the presence of a disease or species can be determined by impact factors [38]. 

However, it is still a challenge to compare the relative performance among different models and ultimately determine the best modeling strategy [39]. Most studies focus on single-model predictions now [24,40], and even the few that include comparisons of several algorithms also lack further combined modeling [22]. In particular, to the best of our knowledge, studies on *Cx. tritaeniorhynchus* are dominated by maximum entropy models (MAXENT) or general boosted models (GBM) with the lack of multiple algorithms [7,24], pointing to the possible room for more exploration in modeling technology. Whilst various algorithms have been applied in other species [41], we still can further use an ensemble forecast approach accounting for the variability among various algorithms to obtain the central tendency [42]. Theoretically, the ensemble forecast is a meta-algorithm that combines several modeling techniques into a single predictive model to decrease variance and bias and improve prediction significantly [42,43].

In this study, we used ensemble modeling techniques that combined ten different models to map the global distribution of *Cx. tritaeniorhynchus* and assessed the impact of two climate change scenarios on the distribution using vector habitat suitability maps. To the best of our knowledge, this is the first prediction study of the current and future global distribution of *Cx. tritaeniorhynchus*, and to some extent, this study explored the most comprehensive impact factors and applied a rich variety of algorithms. The identification of the risk areas of *Cx. tritaeniorhynchus* would help in providing guidelines to formulate vector control strategies globally.

## 2. Materials and Methods

### 2.1. Occurrence Data

We conducted an extensive literature search using the keywords “*Culex Tritaeniorhynchus*” and “*Cx. tritaeniorhynchus*” in the PubMed, ScienceDirect and China National Knowledge Infrastructure (CNKI) before 2021 August, and a comprehensive literature review was developed (Table S1). We collected all occurrence records from the comprehensive literature review and Global Biodiversity Information Facility database (GBIF, http://www.gbif.org/, last accessed on 1 August 2021). A database was created of 2176 occurrences of *Cx. tritaeniorhynchus* for the period 1905–2020. All occurrences were available from online databases to ensure consistency and reproducibility [44,45]. 

We extracted all available location information for each occurrence. Google Earth (https://www.google.com/earth/ (accessed on 1 September 2021) was used to acquire the coordinates of mosquito collection points when the information was not provided. The occurrences with missing geospatial information and absent variable layers were excluded. Duplicate records were deleted, and only one presence point was retained per 2.5 arc–min resolution grid (a 5 × 5 km area) to reduce spatial autocorrelation and avoid inflated accuracy [20,46,47]. In addition, in de-duplication, we gave priority to retaining occurrences close to the time of impact factors. Finally, older occurrences were removed after checking.

### 2.2. Impact Factors

We obtained bioclimatic data, elevation data, vegetation index, pig density, cattle density and human footprint (Table 1). Nineteen bioclimatic variables (1970–2000) with a spatial resolution of 2.5 arc–min were from the latest WorldClim version 2.1 (Fick, S.E. and R.J. Hijmans, Davis, CA, USA) [48], and these variables have been widely applied to model the ecological niche and potential distribution of species.

Altitude as a geographical factor was extracted from elevation data from the Global Land One-km Base Elevation Project (GLOBE) [49]. The Normalized Difference Vegetation Index (NDVI) is a measure of photosynthetic activity, reflecting the spatial and temporal dynamics of vegetation that influences mosquito reproduction and development [50]. We obtained the NDVI data from The VEGETATION Programme. Human footprint was accessed through the global human footprint layer version 2 from Wildlife Conservation Society (WCS), and Center for International Earth Science Information Network (CIESIN) (NASA Socioeconomic Data and Applications Center (SEDAC), Palisades, USA). Human footprint calculates human influence globally, with datasets representing human population density, land transformation, human access and infrastructures [23]. Studies use human footprint as a factor in assessing human impact on species [29,51]. In addition, we obtained the pig and cattle density from the Livestock Geo-Wiki.

The WorldClim dataset also offers the future distribution of bioclimatic variables based on different models such as MIROC5, MIROC6 and GCMs. [28]. MIROC6 is the latest version of MIROC5 from CMIP6, with good performance in simulating mean climate, internal climate variability and climate sensitivity [52], and simulates extreme and summer precipitation better than other GCMs, especially in South Asia [53]. Thus, we used the future bioclimatic variables from MIROC6 to model the distribution of *Cx. tritaeniorhynchus* in the future. These are a new set of emission scenarios driven by different socioeconomic assumptions developed by the energy modeling community in MIROC6. Two extremes for the Shared Socioeconomic Pathways (SSPs), SSP5-8.5 and SSP1-2.6, respectively, which represent the most severe and the least emission scenarios [54], were applied in modeling. We used corresponding type future climate variables under SSP5-8.5 and SSP1-2.6 scenarios in four periods (2021–2040, 2041–2060, 2061–2080 and 2081–2100) and the other non-climatic variables above to model future projections. Finally, we obtained eight future distributions for each period and scenario.

The same resolution was used for all variables using resampling technique, and the masking technique was applied to extract the same geographic extent of all maps using ArcGIS 10.2 (ESRI Inc., Redlands, CA, USA). To avoid the effects of overparameterization and multicollinearity among variables, we excluded variables with a high intercorrelation using Pearson’s correlation coefficient (r > 0.70) (Figure S1). Finally, 13 variables were selected as impact factors in modeling (Table 1), rather than the original 24 variables.

### 2.3. Model Development and Assessment

Ensemble species distribution modeling using the biomod2 package in R 4.1.0 (The R Foundation for Statistical Computing, Topeka, KS, USA) was used to predict the distribution of *Cx. tritaeniorhynchus*. We implemented ten different algorithms: general linear models (GLMs), general boosted models (GBMs, also referred to as boosted regression trees), general additive models (GAMs), classification tree analysis (CTA), artificial neural networks (ANNs), surface range envelope (SRE), flexible discriminant analysis (FDA), multiple adaptive regression splines (MARS), random forests (RFs) and maximum entropy (MAXENT) [43]. Several models require both absence and present records, and we introduced 667 pseudo-absence records using random extraction and had 1 334 unique points in the models.

All the presence and absence points were randomly split into two parts, with 75% of the data to train and 25% to test the models. To synthetically evaluate and compare the performances of models, we used the receiver operating characteristic curve (area under the curve, AUC) and True Skill Statistics (TSS), which were generally applied in predictive species distribution [26]. These two methods are independent, but it is desirable to execute both methods for comprehensive assessment [43]. Low values reflected a model that is no better than a random association between species presence/absence and underlying variables, and high values close to 1.0 reflect a strong signal of association, indicating excellent model performance [55].

We performed three evaluation runs during the modeling and obtained a total of 30 models (10 modeling methods ×3), and average values of testing AUC and TSS were measured [26]. For ensemble modeling, only the models with TSS > 0.8 and AUC > 0.9 were considered [26,55], and the consensus method based on the weighted mean approach increased the model accuracy [56]. We calculated the ranks for the importance of effective variables to compare their predictability among the models. Uncertainty was calculated as the coefficient of variation of the projections between the individual models in the ensemble, reflecting the disagreement between SDM techniques [57]. The agreement between techniques was not a direct indication of accuracy but only an addition to our results (Figures S2 and S3). With regard to the urgency of the control and the assessment of suitability for other species [28,41], we also developed geographical distribution maps with five levels according to the probability of occurrence.

## 3. Results

### 3.1. Overall Occurrences

**Figure 1:**
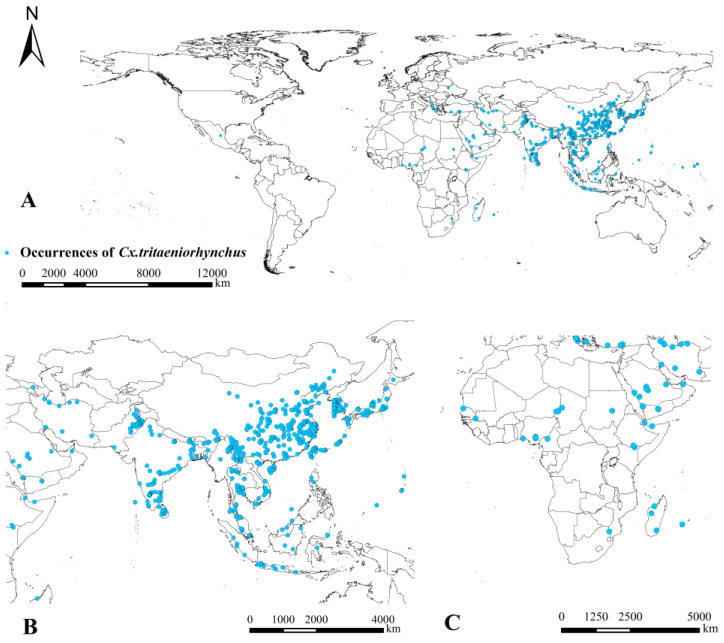
Geographical distribution of *Cx. tritaeniorhynchus* occurrence records. The map showed unique occurrences in (**A**) all world, (**B**) Asia and (**C**) Africa.

Among 2176 occurrences for the period 1905–2020, there were 914 duplicated occurrences, 422 occurrences without geospatial information, and 173 occurrences restricted by spatial resolution, which were removed from our database. For the remaining 667 spatially unique occurrences (Table S2), *Cx. tritaeniorhynchus* had been detected in 41 countries from 5 continents, including 22 Asian countries (90%) and 19 other countries (10%). Most occurrences were in China (43.0%), followed by India (11.4%), Japan (7.0%), Thailand (6.9%), Korea (6.3%) and Pakistan (5.4%) (Figure 1). 

### 3.2. Model Evaluation

**Figure 2:**
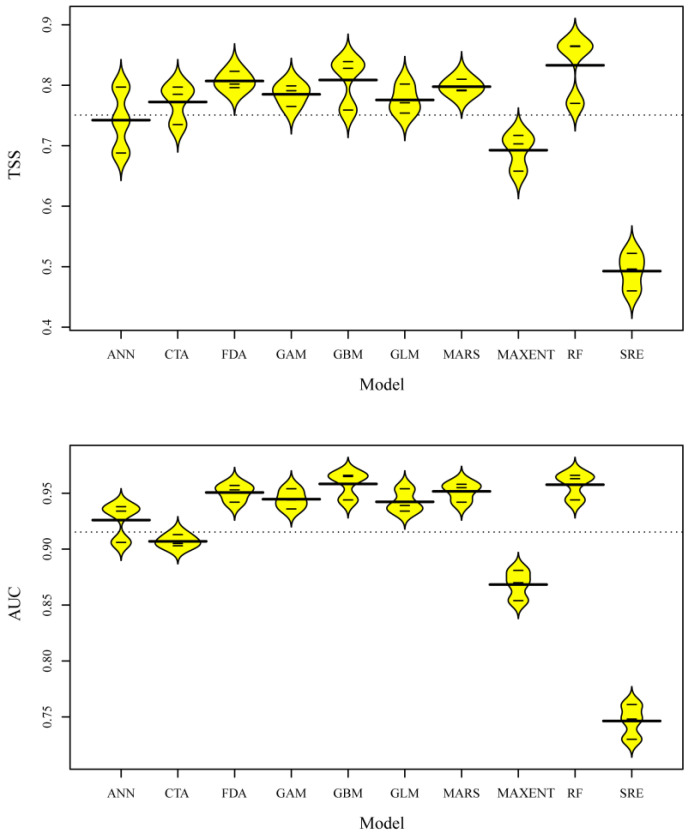
Beanplot illustrating performance in terms of TSS and AUC values over the 30 predictive models (10 algorithms × 3 runs). The bold horizontal lines indicate the overall average values for each algorithm. GLM, general linear model; GBM, general boosted model; GAM, general additive model; CTA, classification tree analysis; ANN, artificial neural network; SRE, surface range envelope; FDA, flexible discriminant analysis; MARS, multiple adaptive regression spline; RF, random forest; MAXENT, maximum entropy.

Figure 2 shows the predictability of ten algorithms. RF (TSS = 0.833, AUC = 0.958) performed the best, while SRE (TSS = 0.493, AUC = 0.746) performed the worst. We obtained a final ensemble model by incorporating weighted runs from the eight models with TSS > 0.8 and AUC > 0.9 (two using GBM, FDA and RF, and one using MARS and GLM). Compared to any single model, the performance of the ensemble model showed the best predictability (TSS = 0.864 and AUC = 0.982). Thus, we used the final ensemble model to predict current and future distributions.

### 3.3. Variable Importance

**Figure 3:**
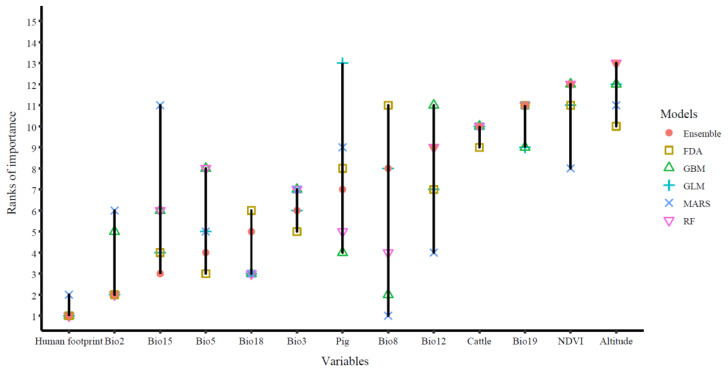
Ranks of the importance according to the contribution of each variable in different algorithms (RF, GBM, GLM, FDA and MARS) and the final ensemble model. The length of the vertical bars indicate the difference in the rank of importance for the same variable in the above algorithms. The lower rank values indicated higher importance. RF, random forest; GBM, general boosted model; GLM, general linear model; FDA, flexible discriminant analysis; MARS, multiple adaptive regression spline.

Figure 3 ranked the importance of the 13 impact factors. The final ensemble model indicated that human footprint was the most important factor for the occurrence of *Cx. tritaeniorhynchus*. For other algorithms, human footprint was also often at the top. In contrast, altitude was often at the bottom of the ranks. Bio8, pig and bio15 ranked differently for various models with a greater length of the vertical bars. Human footprint and cattle performed similarly for different algorithms. 

### 3.4. Predicted Current Geographical Distribution of Cx. tritaeniorhynchus

**Figure 4:**
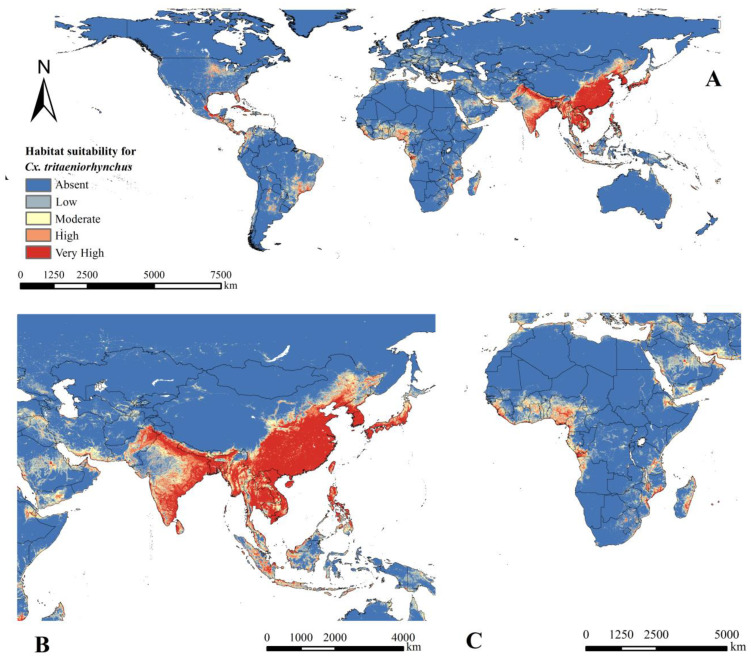
Predicted current geographical distribution of *Cx. tritaeniorhynchus.* The habitat suitability of geographical distribution was divided into five levels according to the probability of occurrence for *Cx. tritaeniorhynchus* as follows: absent: 0–0.2, low: 0.2–0.4, moderate: 0.4–0.6, high: 0.6–0.8 and very high: 0.8–1.0. In addition the closer to 0, the lower the probability of *Cx. tritaeniorhynchus* occurring. The closer to 1, the higher the probability of *Cx. tritaeniorhynchus* occurring. (**A**) All world, (**B**) Asia and (**C**) Africa.

**Figure 5:**
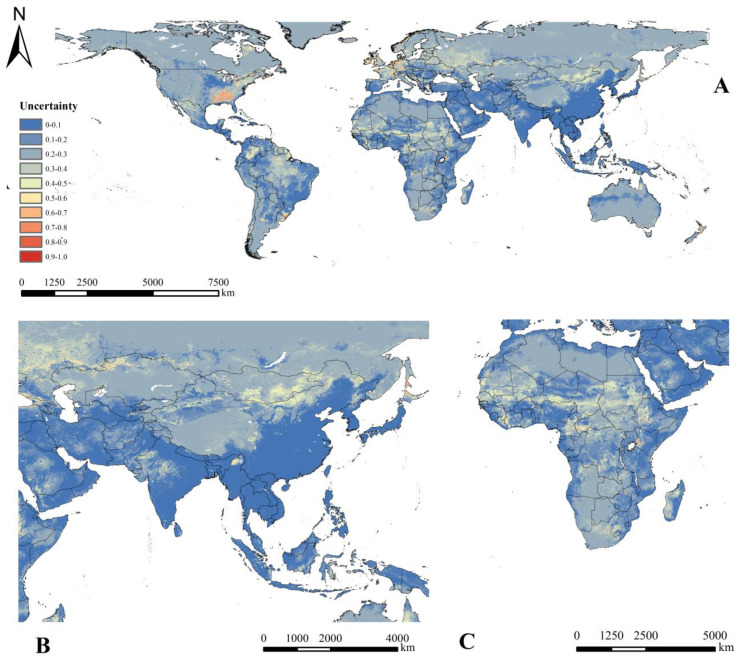
Current uncertainty of *Cx. tritaeniorhynchus*. (**A**) All world, (**B**) Asia and (**C**) Africa.

The predicted distribution map according to the final ensemble model indicated that *Cx. tritaeniorhynchus* was mainly distributed in the tropics and subtropics (Figure 4). Asia showed the largest area with high habitat suitability for *Cx. tritaeniorhynchus*, including all southeastern Asian countries such as China, India, Japan, Myanmar, Thailand, Laos, Vietnam, Bangladesh and Cambodia. High habitat suitability was also found in countries on other continents such as Central America, Mexico, Cuba, Colombia, western Norway, northern Greece, Nigeria and Tanzania. The region of highest uncertainty was in North America, with scattered areas of high uncertainty in Western Europe, where relatively clear inconsistencies were observed between the individual models (Figure 5).

### 3.5. Predicted Future Geographical Distribution of Cx. tritaeniorhynchus

**Figure 6:**
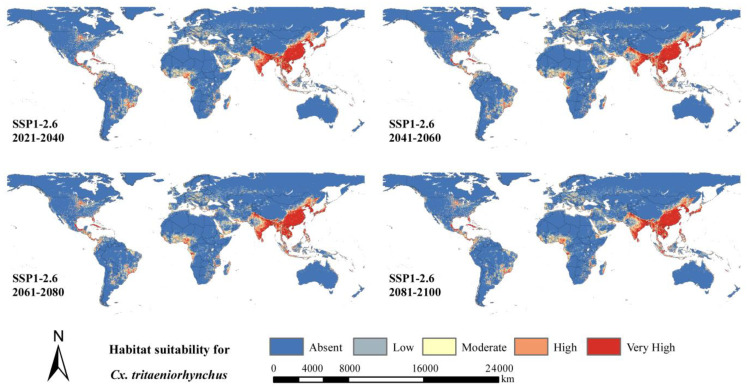
Predicted future geographical distribution of *Cx. tritaeniorhynchus* under SSP1-2.6 scenario in four periods (2021–2040, 2041–2060, 2061–2080 and 2081–2100).

**Figure 7:**
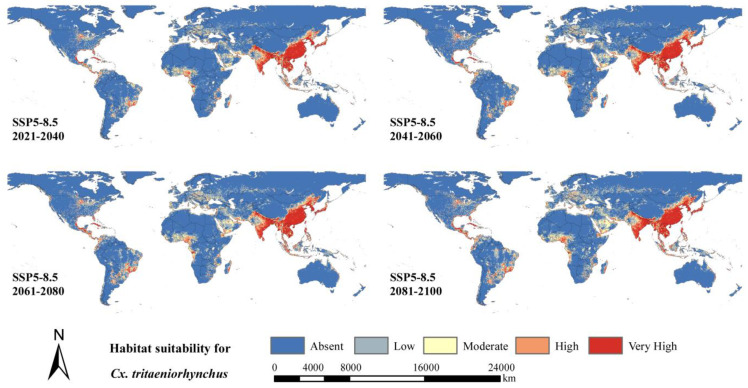
Predicted future geographical distribution of *Cx. tritaeniorhynchus* under SSP5-8.5 scenario in four periods (2021–2040, 2041–2060, 2061–2080 and 2081–2100).

Figure 6 and Figure 7 shows the predicted future distribution of *Cx. tritaeniorhynchus* under two extreme emission scenarios. High habitat suitability would still be distributed in southeastern Asia, Central Africa, southeastern North America and eastern South America. However, the levels of habitat suitability would increase in northern China, Japan, Indonesia, Iraq, Central Saudi Arabia, eastern Brazil, the United States and various countries in Western Europe, even under the SSP1-2.6 scenario with the lowest emissions. In addition to these areas, under the SSP5-8.5 scenario with the highest emissions, the levels of habitat suitability would increase more markedly in other areas, including Central India, Korea, eastern Russia, southern Niger, northern Argentina, Bolivia and southern Venezuela. Meanwhile, there was no significant overall change of uncertainty for SSP1-2.6 (Figure S2). For Figure S3, there was a global increase for the areas with already some obvious uncertainties in the future. Especially for North America and Europe, the uncertainty of high growth was worth noting.

Table 2 shows that under the SSP1-2.6 scenario, the areas with low, moderate, high and very high habitat suitability levels would show the largest expansion in 2041–2060 and then decline slightly afterward in 2061–2080 and 2081–2100. In addition, the area of low habitat suitability level would increase to 1150 km^2^ in 2041–2060 from 983 km^2^ at present. Under the SSP5-8.5 scenario, there would be a significantly greater expansion of habitat suitability with low, moderate, high and very high levels, which was predicted to peak at the end of the 21st century. The area of low suitability habitat for *Cx. tritaeniorhynchus* would increase the most in 2081–2100 to approximately 1451 km^2^. The area would be 20 to 30% larger for moderate, high and very high habitat suitability compared to the current distribution. An extension was observed in the future along the coasts and river systems such as the Amazon River, Congo River and Danube River.

## 4. Discussion

The study provided the most comprehensive maps for predicted global *Cx. tritaeniorhynchus* distribution at present. We also initially predicted the future global distribution under the SSP5-8.5 and SSP1-2.6 scenarios. We developed an ensemble model (TSS = 0.864 and AUC = 0.982) with various algorithms and broader impact factors, such as climate, altitude, NDVI, human footprint, pig density and cattle density. The results provided important information to develop targeted measures for *Cx. tritaeniorhynchus* monitoring.

The high suitable areas of *Cx. tritaeniorhynchus* were in the tropics and subtropics. In our maps, we found obvious high habitat suitability in Asia, which was similar to previous studies [7]. Further, when the projections were first extended globally in our study, a tendency of *Cx. tritaeniorhynchus* to spread from the coastlines to the inlands was observed on all continents. The possible ecological reasons are that water plays an important role in larval development, and summer monsoon winds further help mosquitoes spread and migrate. It is also interpreted as the effects of temperature changes caused by topography and land/sea contrasts [58]. A similar trend was observed inland. For example, the mosquito habitat is mainly distributed along the river system, including the Congo River in Africa, the Danube River in Europe and the Paraguay River in South America (Figure 4). As water systems are often accompanied by large populations, the surrounding should be the focus of future surveillance. In addition, based on the predicted distribution of *Cx. tritaeniorhynchus,* we grouped countries into five habitat suitability levels in our maps. Specifically, for countries with poor entomological reporting, these classification maps can be used to prioritize surveillance for prevention. This will be especially important in areas not located in Asia where JEV was initially detected or with unprecedented emergence in recent years, such as Italy, Australia and Angola [33,59,60,61]. Beyond the traditional risk zone of *Cx. tritaeniorhynchus* in Asia, our findings filled a gap in the worldwide predictions and provided surveillance guidance on a global scale.

Our study indicated that there would be an obvious expansion of distribution under two different climate models (Figure 6 and Figure 7). Asia would still be the main epidemic area for *Cx. tritaeniorhynchus,* and Europe, North America and Africa would have a larger extension. Considering that the socioeconomic conditions and public health infrastructure in North America and Europe may limit the outbreak and impact of *Cx. tritaeniorhynchus* [62] and that Asia is a traditional epidemic area with rich experience in prevention and control [63], the focus of future studies should shift toward Africa and South America. 

In addition, an extension was observed in the future along the coasts and river systems, and the levels of habitat suitability would increase, to a large degree, related to increasing near-surface temperatures in winter [64]. Compared with the decreases of other mosquito species in the future in northern South America, southern Europe, Central Africa and Southeast Asia [20,65], *Cx. tritaeniorhynchus* is believed to gain a great advantage in mosquito competition due to climate change. Changing conditions in the tropics are more beneficial for the invasion and establishment of *Cx. tritaeniorhynchus* than *Ae. albopictus* as native species.

Multiple variables were related to the distribution of *Cx. tritaeniorhynchus,* and among them, human footprint was the most important one. Similar results have been reported in previous studies [24,29]. Human population characteristics may mediate interactions among mosquito species [66], their hosts [19] and their pathogens [67]. Pigs and cattle played a less important role than human footprint, though *Cx. tritaeniorhynchus* may have an innate feeding preference for pigs and cattle than humans [68]. A possible reason is that human footprint not only represents enough food resources but also extends the living space geographically on account of the large sphere of human activity in recent years.

Bioclimate variables also played an important role in the geographical distribution of *Cx. tritaeniorhynchus*. Bio2, an index of temperature, was the top variable, after human footprint, affecting mosquito distribution. A field survey in India found a strong correlation between temperature and *Cx. tritaeniorhynchus* distribution [69]. Mosquito population, viral species, and temperature also significantly influence both vector competence and vectorial capacity [70]. Bio15, as an indication of precipitation, also affected the distribution of *Cx. tritaeniorhynchus*, and it might improve the condition for larval development and adult survival [71]. Water depth could influence the choice of *Cx. tritaeniorhynchus* in preferring the breeding habitat of mosquitos [72]. Bio8 ranked at the top in MARS but at the bottom in FDA. Using non-parametric regression and linear discriminant analysis (LDA), FDA is a multigroup non-linear discrimination technique [73], and MARS automatically estimates complex non-linear relations based on a series of spline functions of the predictor variables with a two-stage process [74]. With the change of algorithms, the contribution of each variable in modeling has a certain elasticity.

The ensemble model performed best in our study (TSS = 0.864 and AUC = 0.982); the model was statistically robust, and its predictive precision was excellent. For a single model, RF showed the best performance. Although MAXENT has been used alone in some studies to predict *Cx. tritaeniorhynchus* [24], the performance is not relatively ideal among the algorithms in our study. Further, studies have shown that no single model performed well under various conditions [28,75,76,77], which affects the effectiveness of model predictions in application [42,78]. Indeed, the results of the application of the traditional method that selected the best model from multiple models often differ from the actual observations, especially when the observation set is spatially or temporally independent from the calibration set [79,80]. The ensemble modeling technique was applied to deal with inter-model uncertainty well [43,81], and more robust and accurate prediction results were obtained. It is similar to the results from Xia et al. [41], who proposed that the complex relationships that exist between species and variables can explain the general performance of classical models.

There are several limitations of our study. We included a number of impact factors but did not consider the effects of socioeconomic development and anthropogenic changes to the land. The raw data differed in sampling and resolution and might have certain spatial autocorrelation. In addition, we only considered the presence and absence of *Cx. tritaeniorhynchus*, but its abundance, which plays a crucial role in disease transmission [20], was not investigated. Uncertainty existed in these occurrences. The level of mosquito surveillance and the number of scientific reports in different countries influenced the uncertainty of occurrences that we collected from public databases. For the future projection, different selections for the combination of emission and socioeconomic scenarios could result in various results [65]. In the current study, we chose two scenarios representing the lowest and highest emission scenarios in the future for interval coverage. An additional limitation is that the two scenarios used for future projection are only from a single model, viz. MIROC6. In addition, due to the lack of available variables such as NDVI, human footprint, pig and cattle in the future, only future climate variables were added, and other variables remained the same in the future distributions.

## 5. Conclusions

Our study predicted the global geographic distribution of *Cx. tritaeniorhynchus* at different habitat suitability levels. In addition, *Cx. tritaeniorhynchus* is predicted to show a wider distribution in all continents, especially in Western Europe and South America in the future under two extreme emission scenarios (SSP5-8.5 and SSP1-2.6). Targeted strategies for the control and prevention of *Cx. tritaeniorhynchus* should be further strengthened.

## Figures and Tables

**Table 1:** Variables used in the ecological niche modeling.

Variables	Description	Sources
Bio2	Mean diurnal temperature range	WorldClim database (http://www.worldclim.org/ (accessed on 31 August 2021)
Bio3	Isothermality	
Bio5	Max temperature of warmest month	
Bio8	Mean temperature of wettest quarter	
Bio12	Annual precipitation	
Bio15	Precipitation seasonality	
Bio18	Precipitation of warmest quarter	
Bio19	Precipitation of coldest quarter	
Altitude	Altitude from DEM	Global Land One-km Base Elevation Project (GLOBE) (https://www.ngdc.noaa.gov/mgg/topo/globe.html (accessed on 31 August 2021)
NDVI	SPOT VEGETATION Images from spectral bands (SWIR, NIR, RED, BLUE).	https://www.vito-eodata.be/ (accessed on 31 August 2021)
Human footprint	Anthropogenic impacts on the environment	Wildlife Conservation Society (WCS) and Center for International Earth Science Information Network (CIESIN) (http://sedac.ciesin.columbia.edu/data/collection/wildareas-v2 (accessed on 31 August 2021)
Pig	Global livestock distribution	Global pig density grid data (http://www.geo-wiki.org (accessed on 31 August 2021)
Cattle	Global livestock distribution	Global cattle density grid data (http://www.geo-wiki.org (accessed on 31 August 2021)

**Table 2:** Projected future area of different habitat suitability for *Cx. tritaeniorhynchus* under the SSP5-8.5 and SSP1-2.6 scenarios in four periods (2021–2040, 2041–2060, 2061–2080 and 2081–2100).

Scenario	Period	Area of Different Habitat Suitability/Km^2^ (%)				
		Absent	Low	Moderate	High	Very High
	Current	11,041 (81.8)	983 (7.3)	513 (3.8)	377 (2.8)	586 (4.3)
SSP1-2.6	2021–2040	10,711 (79.3)	1129 (8.4)	584 (4.3)	436 (3.2)	640 (4.7)
	2041–2060	10,662 (79.0)	1150 (8.5)	595 (4.4)	444 (3.3)	650 (4.8)
	2061–2080	10,694 (79.2)	1134 (8.4)	587 (4.3)	446 (3.3)	639 (4.7)
	2081–2100	10,733 (79.5)	1129 (8.4)	573 (4.2)	435 (3.2)	630 (4.7)
SSP5-8.5	2021–2040	10,692 (79.2)	1135 (8.4)	594 (4.4)	439 (3.3)	640 (4.7)
	2041–2060	10,507 (77.8)	1218 (9.0)	625 (4.6)	476 (3.5)	674 (5.0)
	2061–2080	10,279 (76.1)	1321 (9.8)	684 (5.1)	512 (3.8)	703 (5.2)
	2081–2100	9995 (74.0)	1451 (10.7)	752 (5.6)	553 (4.1)	749 (5.5)

Note: The habitat suitability of geographical distribution was divided into five levels according to the probability of occurrence for *Cx. tritaeniorhynchus* as follows: absent: 0–0.2, low: 0.2–0.4, moderate: 0.4–0.6, high: 0.6–0.8 and very high: 0.8–1.0. In addition, 0 was completely impossible, and 1.0 was certain for *Cx. tritaeniorhynchus* to occur. The global land area is 135 million square kilometers except for Antarctica. The coverage was differentiated for current and future distributions according to the different periods of climatic variables from WorldClim.

## Data Availability

Data for statistical analysis are available in the Supplementary Materials.

## Supplementary Materials

The following supporting information can be downloaded at: https://www.mdpi.com/article/10.3390/ijerph20064701/s1, Table S1. A comprehensive literature review; Table S2. The final spatially unique occurrences of *Cx. tritaeniorhynchus* in modeling; Figure S1. Correlation coefficients for all variables; Figure S2. Uncertainty of *Cx. tritaeniorhynchus* under SSP1-2.6 scenario in four periods (2021–2040, 2041–2060, 2061–2080 and 2081–2100); Figure S3. Uncertainty of *Cx. tritaeniorhynchus* under SSP5-8.5 scenario in four periods (2021–2040, 2041–2060, 2061–2080 and 2081–2100).
