# Supplementary figures and images for: Global Distribution of Culex tritaeniorhynchus and Impact Factors
Source: Int J Environ Res Public Health. 2023 Mar 7;20(6):4701. doi: 10.3390/ijerph20064701 (PMC10048298; doi:10.3390/ijerph20064701)

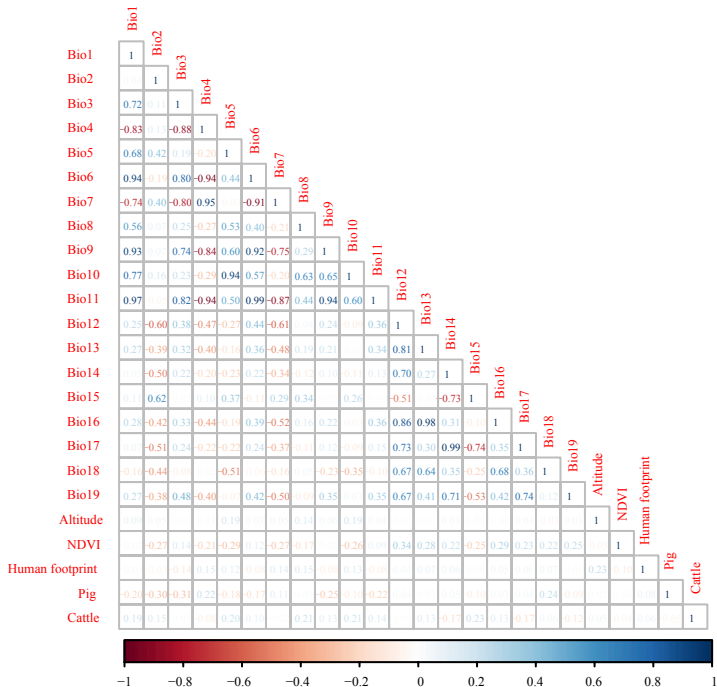

Figure S1. Correlation coefficients for all variables.

Supplement: Supplementary file 1 [file ijerph-20-04701-s001.zip › Figure S1.pdf]

Figure S2. Uncertainty of *Cx. tritaeniorhynchus* under SSP1-2.6 scenario in four periods.

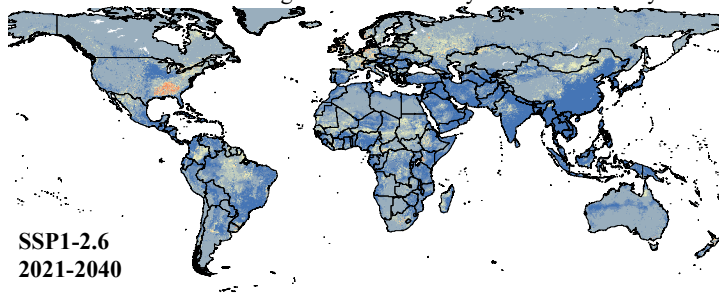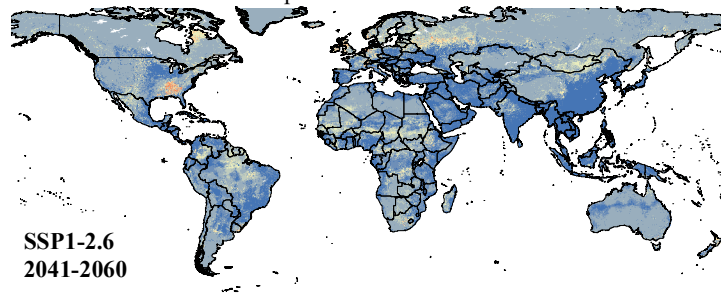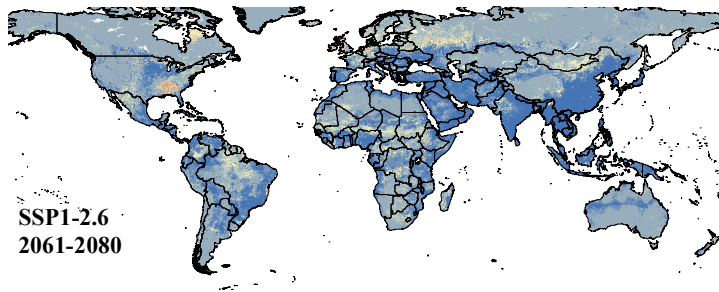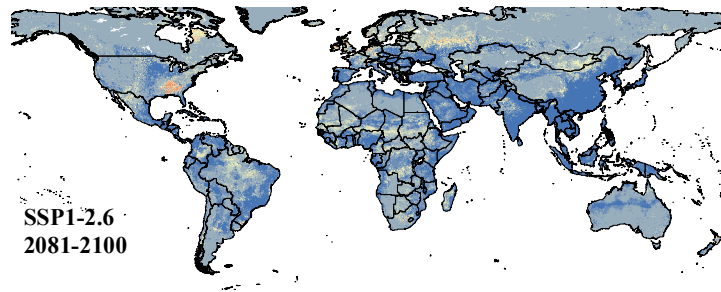

**Uncertainty**

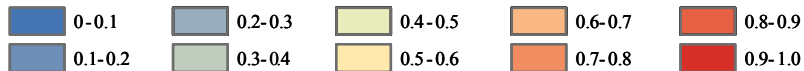

Supplement: Supplementary file 1 [file ijerph-20-04701-s001.zip › Figure S2.pdf]

Figure S3. Uncertainty of *Cx. tritaeniorhynchus* under SSP5-8.5 scenario in four periods.

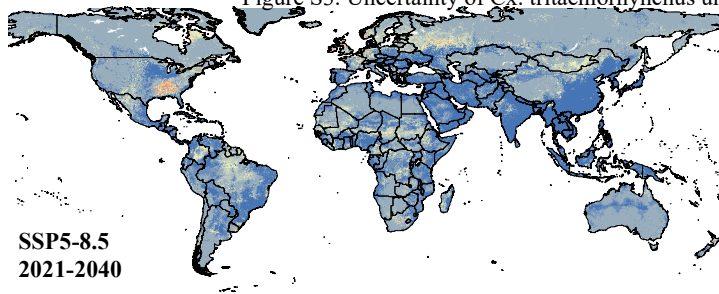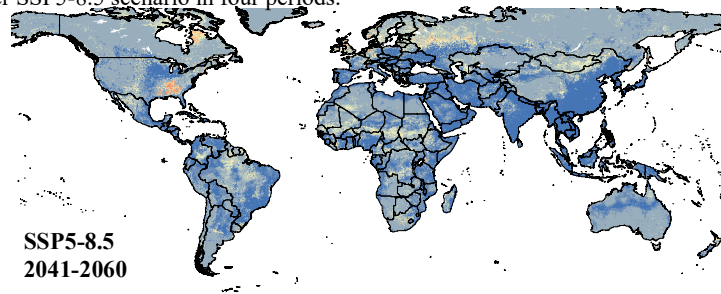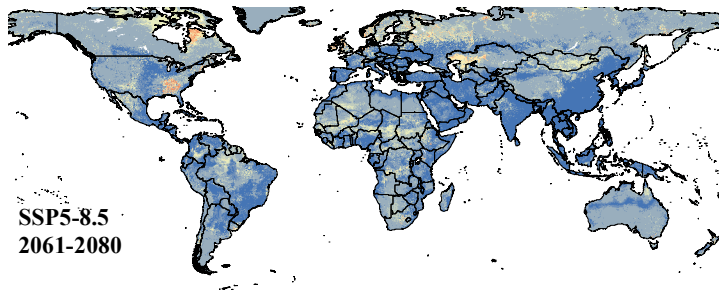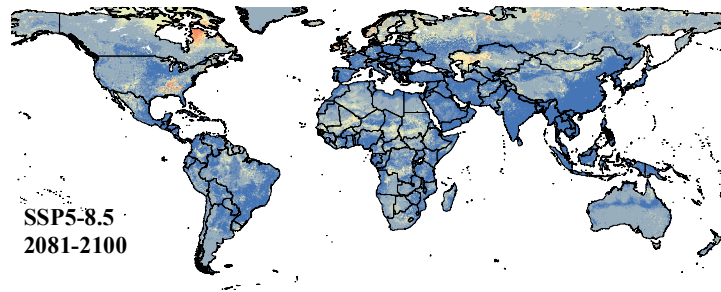

**Uncertainty**

0-0.1

0.2-0.3

0.4-0.5

0.6-0.7

0.8-0.9

0.1-0.2

0.3-0.4

0.5-0.6

0.7-0.8

0.9-1.0

Supplement: Supplementary file 1 [file ijerph-20-04701-s001.zip › Figure S3.pdf]
